# MULTIFAMILY GROUPS DIFFERENT LOOKS: GROUPANALYTICS OPERATIONAL CONCEPTION INTERFAMILY (T. of attachment and open dialogue)

**DOI:** 10.1192/j.eurpsy.2023.2175

**Published:** 2023-07-19

**Authors:** B. Gamo, S. M. Bañon González

**Affiliations:** 1 Infanta Sofía Hospital, Madrid, Spain

## Abstract

**Introduction:**

In this work, I will try to approach birth and growth from my stay in therapy groups, including psychotherapy in multi-family groups from a simple observer, to a driver, alone and in co-therapy, to a member of the experience, to a driver member.

**Objectives:**

Explain why we are interested in our work center in multifamily therapy groups

**Methods:**

Qualitative and narrative of the course of my experience in group therapy over 25 years

**Results:**

Based on my experience, I do not see any probability that coordination/direction will be achieved at the same time as symmetry in participation. Knowledge is power, said Foucault, so I must divest myself of my knowledge in order not to have power, and that power be executed by each of the participants with her own life.

**Conclusions:**

It is in this process that I have been going through, it makes me think to what extent one makes the effort, I have made the effort not to be in the place of excluding the other, the other sick, of segregating him, as if to feel that I have a place of healing, the other must be someone who is the object of being healed. We have the possession of knowledge, to give light to others, in this disciplinary society, of disciplining in prison institutions, as Foucault would say, prisons, hospitals, army.

In this group work, multi-family, we must go to the singularity of the participants, to make them stand out, to get out and overcome the need for those relatives, who prevent them from growing and thawing, and roughly, hold on and take out the healthy virtuality , but that does not mean that we will achieve, even if we get out of the stereotyping and the sickening circle, that we have arrived at what is healthy, because in some way it will end up being just the way in which we look at what is healthy.

In this process, not only do the participants change, but we do too, because otherwise, we would be in a stereotype, regarding our role and the institution itself.

The mentally ill is not only a justification of families, but of society itself, and that other, that other excluded, will be determined by the historical period itself, and social context, the sick of today were not those of the past nor will they be those of the future.

Power, as Foucalt would say, entails resistance, and what is our resistance, that is the question I ask myself, resistance is needed so that power can establish itself, think about counter-power, fissures so that things are renewed, and that is actually what is sought in groups, to work from the cracks of the established

**Disclosure of Interest:**

None Declared

